# A case report on efficacy of Abound™ for anti-EGFR antibody-associated skin disorder in metastatic colon cancer

**DOI:** 10.1186/1477-7819-12-35

**Published:** 2014-02-11

**Authors:** Nobuhisa Matsuhashi, Takao Takahashi, Kenichi Nonaka, Kengo Ichikawa, Kazunori Yawata, Toshiyuki Tanahashi, Hisashi Imai, Yoshiyuki Sasaki, Yoshihiro Tanaka, Naoki Okumura, Kazuya Yamaguchi, Shinji Osada, Kazuhiro Yoshida

**Affiliations:** 1 Surgical Oncology, Gifu University School of Medicine, 1-1 Yanagido, Gifu City 501-1194, Japan

**Keywords:** Abound, Anti-EGFR antibody, Skin disorder, Colon cancer

## Abstract

**Background:**

Panitumumab is a full human epidermal growth factor receptor (EGFR) monoclonal antibody, an agent for metastatic colorectal cancer therapy. One of the most general adverse events of anti-EGFR monoclonal antibody therapy is skin disorder. At the present time, although prophylaxis of skin disorder is important for continuation of cancer therapy, there are no effective precautionary treatments.

**Case presentation:**

A 73-year-old male with sigmoid colon cancer and synchronous lung metastasis was treated with panitumumab, an alone anti-EGFR monoclonal antibody as the third-line therapy.

During the nine courses of the therapy, the response was stable disease (SD), but skin disorder gradually appeared obviously (CTCAE version 4.0: Grade 2). After 1 month of administration of Abound™, symptoms of the skin disorder improved (CTCAE version 4.0: Grade 1), thus the antibody therapy could be continued.

**Conclusion:**

We report that Abound™ was apparently effective in the treatment for anti-EGFR antibody-associated skin disorder. In the future, Abound™ could be expected as an agent for skin disorder as one of the side effects of colorectal cancer therapy.

## Background

In recent years, remarkable progress has been made in chemotherapy for colorectal cancer. In particular, the treatment for advanced or metastatic colorectal cancer has dramatically improved owing to the development of FOLFOX and FOLFIRI therapies. Furthermore, the introduction of targeted therapy has made the treatment more effective and helpful for patients suffering from colorectal cancer. However, as an example of peripheral neuropathy, a serious major side effect of oxaliplatin (L-OHP), the control of adverse events is difficult for the continuation of cancer therapy. In addition, at the same time, the prevention of skin disorder associated with anti-epidermal growth factor receptor (EGFR) antibody therapy is important to continue the cancer therapy. However, at present, treatment associated with the skin disorder is mainly symptomatic.

Abound™ (ABBOTT JAPAN CO., LTD, Tokyo) constituted by a mixture of β-hydroxyl β-methylbutyrate, glutamine, and arginine (HMB/Gln/Arg). Abound™ previously showed activity for healing bed ulcers, increasing lean body mass (LBM) among patients with cancer cachexia [1,2]. Therefore, our hypothesis considered whether Abound™ is effective for cancer patients with skin disorder. We report that Abound™ was effective for a non-resectable colorectal cancer patient treated with an anti-EGFR antigen panitumumab who had developed skin disorder.

## Case presentation

A 74-year-old male with sigmoid colon cancer and synchronous lung metastasis (stage IV) underwent high anterior resection and D3 lymphadenectomy. The patient received 16 courses of FOLFOX and bevacizumab (BV) as first-line therapy, postoperatively. For the reason of disease progression, the patient was followed by BV and FOLFIRI as second-line therapy. The patient’s performance status (PS) went down to PS 1 in accordance with accumulation of the side effect of FOLFIRI therapy, but disease control indicated progression of the disease. Therefore, the patient was started on only panitumumab therapy, an anti-EGFR antigen, in order to wild type the Kras gene type.

An antibiotic agent, minocycline hydrochloride (minocycline), and an external preparation, dexamethasone, were administered form the start of the panitumumab therapy for prophylaxis of the skin disorder.

**Figure 1:**
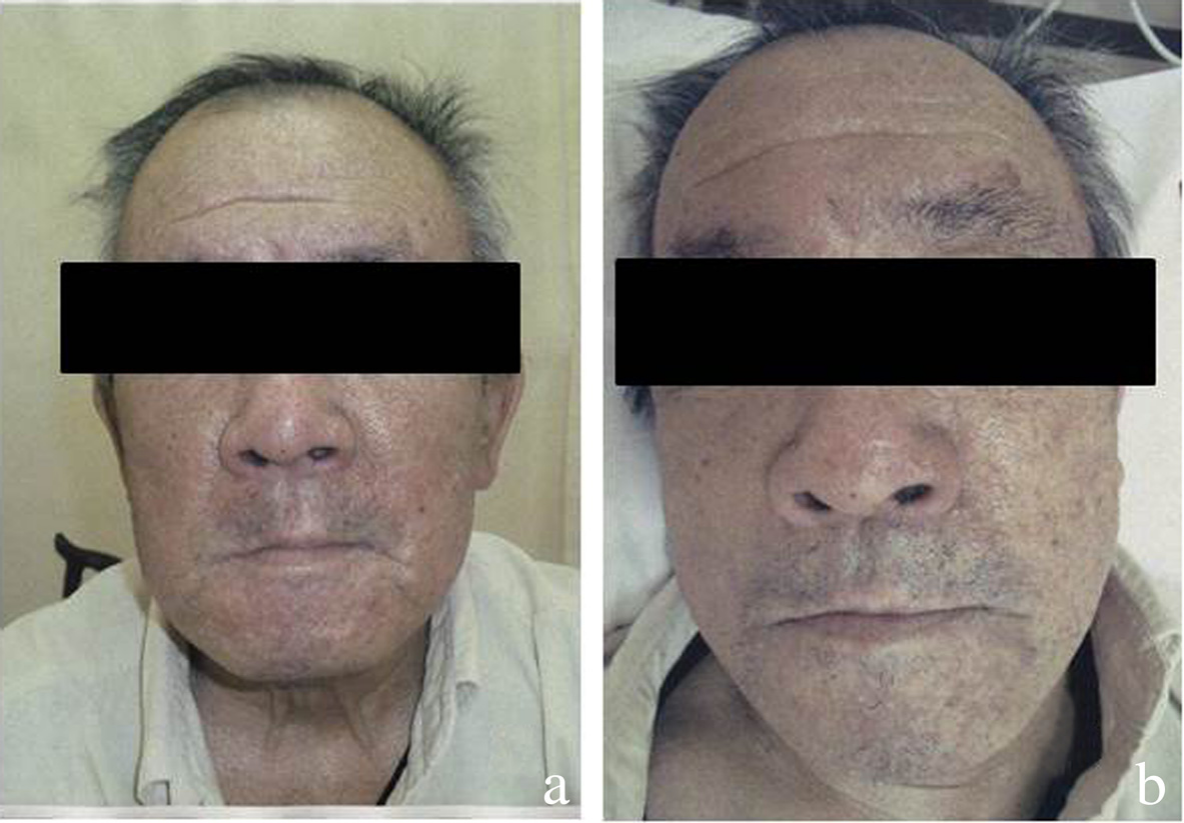
**Before and after images of Abound**^**TM **^**treatment: face. (a)** Before Abound^TM^ was administered (CTCAE version 4.0: Grade 2). **(b)** After Abound^TM^ was administered (CTCAE version 4.0: Grade 1). CTCAE, common terminology criteria for adverse events.

**Figure 2:**
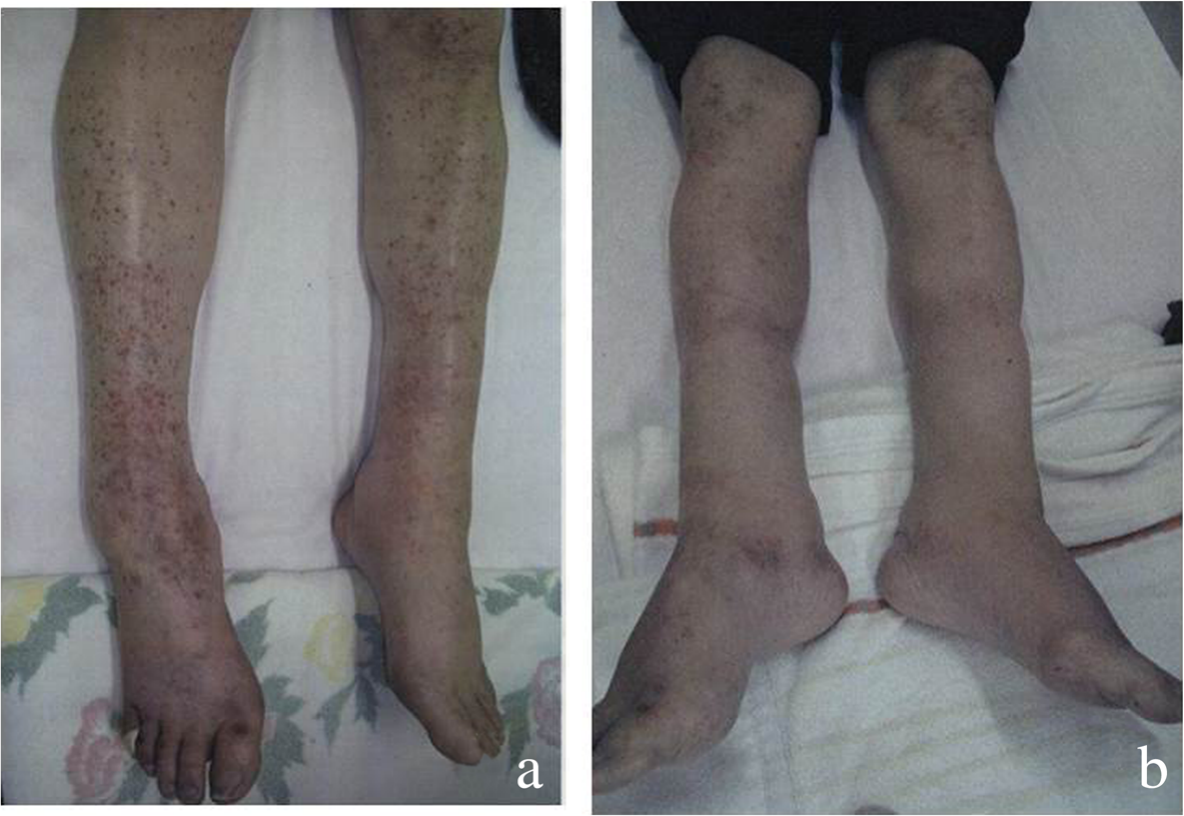
**Before and after images of Abound™ treatment: lower limbs. (a)** Before Abound™ was administered (CTCAE version 4.0: Grade 2). **(b)** After Abound™ was administered (CTCAE version 4.0: Grade 0). CTCAE, common terminology criteria for adverse events.

During the second course of the anti-EGFR antibody therapy, skin disorder appeared on the patient’s facial surfaces and gradually on other parts. The symptomatic treatment was continued; however, at the end of the ninth course of the anti-EGFR antibody therapy, the skin disorder was observed on both the lower limbs as well as on the face remarkably. Thus, Abound^TM^ containing HMB/Gln/Arg was administered with two packs (48 g) a day. The skin disorder on both the lower limbs profoundly improved after 1 month of continuation of Abound™ (Figures 1a,b and 2a,b).

## Discussion

Molecules of the EGFR family compose signal transduction pathways and play a major role in intracellular reaction processes [3-5]. New entities have been developed to target the pathway because EGFR has been observed in high frequency in non-small cell lung cancer (NSCLC) and colorectal or pancreatic cancer. EGFR tyrosine kinase inhibitors, including gefitinib and erlotinib, were launched as chemotherapy agents for NSCLC. Recently, cetuximab and panitumumab as EGFR monoclonal antigens have been made available for the treatment of colorectal cancer [6-9].

These agents are characteristically known to cause the side effect of frequent skin disorder, and thus the control of the skin disorder itself and its symptoms are very important for cancer therapy. From this point of view, skin disorder with inhibitors of the EGFR system can be used to continue the drug administration for cancer therapy, which is most important simultaneous with symptom control of the skin disorder [10]. In addition, with regards to erlotinib, correlation between the severity of skin disorder and the curative effect against cancer has shown that when the severity of skin disorder is higher, the curative effect is higher, and it has been suggested that cetuximab and panitumumab may demonstrate the same characteristics [11,12].

At present, minocycline hydrochloride (minocycline) with steroidal agents are recognized as supportive treatment for the prevention of skin disorder with anti-EGFR antibody, utilized in various institutes, in accordance with the results of the skin toxicity evaluation protocol with panitumumab (STEPP) study [13].

In this case, we administered Abound™ containing HMB/Gln/Arg, and observed improvement of the skin disorder. HMB, a metabolite of leucine, is suggested to promote collagen deposition with modification of muscle protein degradation and to modulate inflammatory reaction by inhibiting the activity of NF-κB. Arginine is a semi-essential amino acid that can enhance immunity and wound healing by potentiating the cellulation process of fibroblasts. Glutamine is supposed to relate to the metabolic response of external injury and sepsis and to enhance gut immunity by affecting lymphocytes and intestinal epithelium cells, and has an effect on the improvement of gastrointestinal disorders for patients receiving anti-cancer chemotherapy. Since Abound^TM^ is a dietary supplement, it is well tolerated, without any reported side effects. Abound™ has been shown to have efficacy on the nutritional management of cancer and HIV cachexia patients, and HMB/Gln/Arg supplementation can be important for those patients [2,14-16]. The HMB/Gln/Arg levels in advanced, metastatic, or recurrent cancer patients are considered to be low. Abound™, one pack of which contains 1,200 mg of HMB, 7,000 mg of glutamine, and 7,000 mg of arginine, can support balanced nutritional management, and in this case report we suggest that Abound™ may promote wound healing in skin disorder.

## Conclusion

In the future, prospective studies of Abound™ in patients treated with anti-EGFR antibody is necessary, but our report indicates that Abound™ may have the potential to support nutritional status and to improve skin disorder. Thus, when larger studies can demonstrate the efficacy, Abound™ could provide an effective option for the management of side effects in colorectal cancer treatment.

## Consent

Written informed consent was obtained from all patients enrolled in the investigation. The study protocol conformed to the ethical guidelines of the 1975 Declaration of Helsinki and the guidelines of the regional ethical committees of Zurich, Switzerland, and Basel, Switzerland.

## Abbreviations

Arg: Arginine; BV: Bevacizumab; CTCAE: Common terminology criteria for adverse events; EGFR: Epidermal growth factor receptor; Gln: Glutamine; HMB: β-hydroxyl β-methylbutyrate; LBM: Lean body mass; L-OHP: Oxaliplatin; NSCLC: Non-small cell lung cancer; PS: Performance status; SD: Stable disease; STEPP: Skin toxicity evaluation protocol with panitumumab.

## Competing interests

The authors declare that they have no competing interests.

## Authors’ contributions

NM and TT conceived the study and design. NM, TT, KN, KI, KY, TT, HI, YS, YT, NO, KY, and SO undertook acquisition of data. NM analyzed and interpreted the data and drafted the manuscript. NM, TT, and KY performed critical revision of the manuscript. KY supervised the study. All authors read and approved the final manuscript.
